# cDNA-AFLP analysis of plant and pathogen genes expressed in grapevine infected with *Plasmopara viticola*

**DOI:** 10.1186/1471-2164-9-142

**Published:** 2008-03-26

**Authors:** Marianna Polesani, Filomena Desario, Alberto Ferrarini, Anita Zamboni, Mario Pezzotti, Andreas Kortekamp, Annalisa Polverari

**Affiliations:** 1 Scientific and Technologic Department, University of Verona, 37134 Verona, Italy; 2 Institute of Special Crop Cultivation and Crop Physiology, University of Hohenheim, 70593 Stuttgart, Germany; 3 Department for Sciences, Technologies e Markets of Grapevine and Wine, 37029 San Floriano di Valpolicella (VR), Italy

## Abstract

**Background:**

The oomycete *Plasmopara viticola *(Berk. and Curt.) Berl. and de Toni causes downy mildew in grapevine (*Vitis vinifera *L.). This pathogen is strictly biotrophic, thus completely dependent on living host cells for its survival. The molecular basis of compatibility and disease development in this system is poorly understood. We have carried out a large-scale cDNA-AFLP analysis to identify grapevine and *P. viticola *genes associated with the infection process.

**Results:**

We carried out cDNA-AFLP analysis on artificially infected leaves of the susceptible cultivar Riesling at the oil spot stage, on water-treated leaves and on a sample of pure sporangia as controls. Selective amplifications with 128 primer combinations allowed the visualization of about 7000 transcript-derived fragments (TDFs) in infected leaves, 1196 of which (17%) were differentially expressed. We sequenced 984 fragments, 804 of which were identified as grapevine transcripts after homology searching, while 96 were homologous to sequences in *Phytophthora *spp. databases and were attributed to *P. viticola*. There were 82 orphan TDFs. Many grapevine genes spanning almost all functional categories were downregulated during infection, especially genes involved in photosynthesis. Grapevine genes homologous to known resistance genes also tended to be repressed, as were several resistance gene analogs and carbonic anhydrase (recently implicated in pathogen resistance). In contrast, genes encoding cytoskeletal components, enzymes of the phenylpropanoid and beta-oxidation pathways, and pathogenesis related proteins were primarily upregulated during infection. The majority of *P. viticola *transcripts expressed *in planta *showed homology to genes of unknown function or to genomic *Phytophthora *sequences, but genes related to metabolism, energy production, transport and signal transduction were also identified.

**Conclusion:**

This study provides the first global catalogue of grapevine and *P. viticola *genes expressed during infection, together with their functional annotations. This will help to elucidate the molecular basis of the infection process and identify genes and chemicals that could help to inhibit the pathogen.

## Background

*Plasmopara viticola *(Berk. and Curt.) Berl. and de Toni is an obligate biotrophic plant pathogen [1] that causes downy mildew, a devastating disease resulting in significant economic losses as well as environmental damage through the repetitive applications of fungicides.

Primary infection begins with over-wintering oospores, which germinate into motile zoospores that can actively locate stomata [2,3] and start the infection process. Colonization involves intercellular mycelial growth and the differentiation of haustoria, which penetrate parenchyma cells by invaginating but not breaking the plasma membrane [4]. This highly specialized nutritional strategy, which typifies biotrophic plant pathogens such as powdery mildews, downy mildews and rusts, probably involves the strict control of host cell metabolism which is diverted to maintain pathogen survival and compatibility [5]. Further infection cycles are initiated through the release of zoosporangia emerging from stomata. The cycles end with the sexual production of over-wintering oospores.

While the epidemiology of the pathogen is understood well enough to generate computer models of epidemics, the molecular aspects of the infection process are largely unknown. The main recognized role of haustoria is to obtain nutritional resources from the plant cell, but the synthesis of additional gene products and metabolites [6,7] suggests that signals are exchanged between the pathogen and host to establish and maintain compatibility and possibly to suppress defense responses [7]. Secreted virulence factors may be involved in this process [8] and four such gene products have recently been identified in other oomycetes [9-12]. Following the recent completion of *Phytophthora *spp. sequencing projects [13], about 700 *avr *homologues have been predicted based on the presence of a signal peptide and a RXRL-EER motif, typical of known cytoplasmic effectors of oomycete pathogens [9,14].

The plant's response to infection has been characterized predominantly through the study of incompatibility in the resistant species *Vitis riparia, V. rupestris *and *Muscadinia rotundifolia*. Attempts have been made to introgress resistance into cultivated *V. vinifera *genotypes [15-18], although the quality and the specific organolectic characteristics of wines are not easy to reproduce in interspecific hybrids. Some of resistance mechanisms have been elucidated [19-22] and they include physical barriers such as hairs and stomatal closure, the accumulation of phenolic antimicrobial compounds, increased peroxidase activity, the accumulation of pathogenesis related proteins and the hypersensitive response [23].

Molecular data from the direct investigation of compatible interactions in cultivated grapevine genotypes is scarce, and indeed downy mildew has received little attention compared to diseases carried by other biotrophic pathogens, such as powdery mildews and rusts. Understanding the basis of susceptibility would greatly assist in the development of new control strategies and the identification of pathogen and host factors required for disease progression.

One useful approach to the molecular analysis of plant-pathogen interactions is the determination of changes in steady state mRNA levels occurring in both the host and the pathogen during infection. Such transcriptomic approaches have been undertaken for different plant-oomycete interactions either by microarray analysis or alternative, open-architecture technologies, thus revealing novel information about pathogen genes [24-29]. A few studies have also included proteomic analysis [30].

The expression of selected grapevine genes during *P. viticola *infection was reported recently [21], but there has been no large-scale analysis and pathogen genomic information is also very scarce (fewer than 30 sequences in GenBank). In this article, we report the results of cDNA-AFLP analysis to identify infection-related transcripts in *P. viticola *and grapevine. Our data show that downy mildew infection of grapevine at the oil spot stage involves the downregulation of many grapevine genes with diverse functions, and the induction of pathogen genes representing important metabolic pathways such as protein synthesis, transport and energy metabolism in infected leaves.

## Results

### cDNA-AFLP analysis

**Figure 1:**
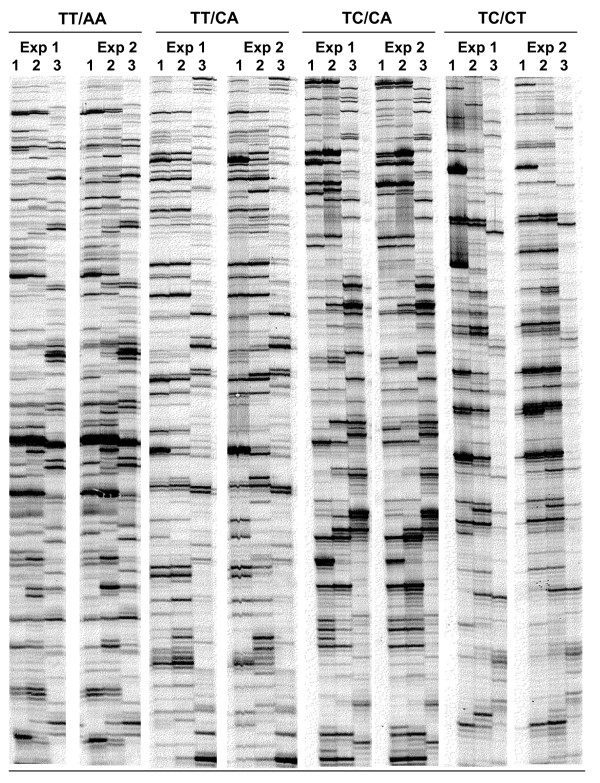
**Expression of grapevine and *P. viticola *transcripts displayed by cDNA-AFLP**. An example showing selective amplification with four different primer combinations, repeated in two biological replicates (Exp. 1 and Exp 2). 1: control water-treated leaves; 2: *P. viticola*-infected leaves; 3: *P. viticola *sporangia.

We carried out a cDNA-AFLP analysis on RNA samples of infected leaves at the oil spot stage, and on healthy control leaves and pure sporangia, as described [31,32]. The oil spot stage was chosen because the compatible interaction is well established and the mycelia produced at this stage are abundant enough to allow the detection of pathogen transcripts, even though the plant cell is still active, since various plant functions are needed to maintain pathogen survival. For each of the 128 primer combinations, 55–75 transcript derived fragments (TDFs) were visualized as bands, 25–760 bp in size, representing approximately 7000 transcripts overall. The same average number of bands per lane was obtained both from grape and from *P. viticola *sporangia samples. To determine the reproducibility of these profiles, the experiments were repeated using additional samples of a biological replicate (Figure 1).

### Detection of differentially expressed transcripts

The infection of grapevine with *P. viticola *resulted in the widespread modulation of steady state mRNA levels. We detected 1196 differentially expressed TDFs, corresponding to about 17% of all visualized transcripts. Each band was excised from the gel, eluted, re-amplified and purified for direct sequencing, yielding 982 cDNA fragments that gave rise to useable sequence data. Among these sequences, 599 were homologous to known expressed grapevine sequences, either as tentative consensus sequences or expressed sequence tags (EST), while 205 were homologous to genomic contigs in the newly released 8.4× Vitis Genome database [33] but were not represented in any EST databases. There were also putative annotations in the UNIPROT database for 72 TDFs, and these were assigned functional categories accordingly. Another 96 TDFs were homologous to known *Phytophthora *spp. sequences derived from the recently completed *Phytophthora *genome sequencing projects [13] and are therefore likely to be *P. viticola *genes expressed during infection. Finally, 82 sequences had no database matches, 65 from TDFs with similar sized bands in the sporangia sample, and 17 expressed uniquely during the infection. Because the grapevine genome is fully sequenced, the 82 additional sequences are likely to represent additional *P. viticola *transcripts with insufficient similarity to known genes in other oomycetes.

### Functional categories of grapevine transcripts modulated by downy mildew infection

**Figure 2:**
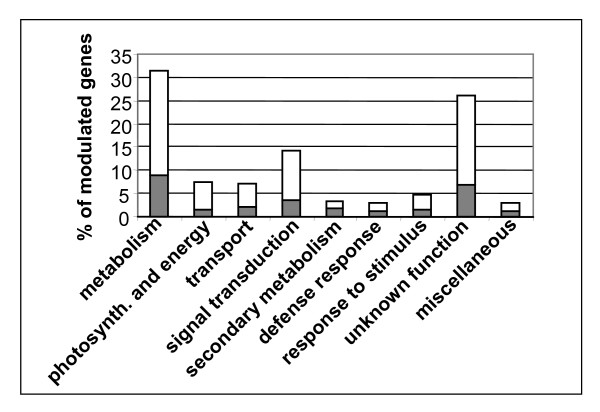
**Grape transcripts modulated by *P. viticola *infection**. Frequency of modulated genes in selected functional categories and percentage of upregulated (grey) or downregulated (white) transcripts, within each category.

A complete list of TDFs isolated from infected grapevine is available in Additional File 1, while a selection of the most interesting TDFs is shown in Table 1. Each transcript was functionally annotated through careful analysis of the scientific literature and the Gene Ontology Database [34]. Figure 2 shows the percentages of grapevine genes assigned to different functional categories. Approximately 31% of the annotated sequences have primary metabolic roles (particularly in protein and carbohydrate metabolism), 14% are involved in signal transduction, and a further 8% in photosynthesis/energy mobilization. Other relevant groups, each accounting for 3–7% of TDFs, include secondary metabolism, cellular transport, defense, and responses to external stimuli. Approximately 26% of the modulated grapevine TDFs corresponded to tentative consensus sequences or ESTs with no known function. Most of the differentially-expressed grapevine transcripts were downregulated during the oil spot stage, and this applied across all but one of the functional categories and was especially prevalent in the photosynthesis/energy mobilization category (~82% down-regulated). The single exception was the secondary metabolism category, where 57% of the differentially-expressed genes were upregulated.

**Table 1:** Selected list of modulated grape transcripts.

**TDF**	**Primer comb**.	**Accession**	**Lenght (bp)**	**I/R**	**Annotation**	Blast score Blastn/Blastx*
**Metabolism**						
192	TC – CA	TC52910	301	**+**	Sucrose synthase (Q9SLS2)	3.12E-63
1627	TT – CT	TC57852	168	**+**	UDP-glucosyltransferase HRA25 (Q9FUJ6)	2.07E-30
245	TA – AC	TC53221	169	**+**	Transketolase, chloroplast precursor (O20250)	1.92E-29
18	TT – AA	TC67193	127	**+**	Raffinose synthase, partial (Q8VWN6)	9.26E-21
1279	CT – TC	TC52362	175	**+**	Fatty acid multifunctional protein (Q9ZPI5)	1.12E-16
134	TC – TT	TC67104	390	**+**	12-oxophytodienoate-10,11-reductase 1 (Q8LAH7)	6.19E-24
993	CA – CA	TC60564	50	**+**	Gibberellin 2-oxidase (Q6TN17)	3.63E-105
215	TT – TA	TC55722	216	**+**	Fatty acid hydroperoxide lyase (Q9AUD8)	5.62E-72
152	TC – TC	Q6X5R6	342	**+**	(Lox2) Lipoxygenase	4.00E-39 *
1036	CC – AT	TC58112	349	**+**	Long-chain acyl-CoA oxidase (O64894)	5.62E-72
1225	CT – AC	TC53311	359	**+**	3-ketoacyl-CoA thiolase (Q6TXD0)	2.10E-09
893	CA – AC	TC67959	104	**+**	3-beta-hydroxy-delta5-steroid dehydrogenase	9.77E-16
347	TA – CG	TC54708	181	**+**	40S ribosomal protein S16 (Q9M5L1)	2.91E-18
1382	CG – TA	TC58494	279	**+**	Ribosomal L10 protein (Q874B2)	2.62E-56
1345	CG – AA	TC51894	289	**+**	Ubiquitin conjugating enzyme E2 (Q42897)	6.56E-55
594	TG – GA	TC60588	186	**+**	Proteasome subunit beta type 5-B precursor (Q9LIP2)	5.35E-37
1493	CT – GG	TC56558	399	**+**	Gamma-glutamylcysteine synthetase (Q6F4I8)	4.64E-35
1293	CT – TG	TC51806	244	**+**	Cysteine synthase (Q43317)	3.08E-25
79	TC – AC	TC68684	457	**+**	Cellulose synthase-like protein D4 (Q8GUZ9)	2.09E-96
1594	TT – TC	TC65238	179	**+**	Tubulin alpha chain, partial (P33629)	4.78E-34
232	TA – AT	TC57434	161	**-**	Nucleotide sugar epimerase-like protein	8.83E-12
1630	TT – CT	Q9ZTP5	105	**-**	Pentose-5-phosphate 3-epimerase	5.85E-10 *
1610	TT – CA	TC54570	167	**-**	Sedoheptulose-bisphosphatase (Q940F8)	1.37E-27
1668	TT – GT	TC54602	412	**-**	Fructose-bisphosphate aldolase (Q6RUF6)	1.47E-50
100	TC – AG	TC54851	224	**-**	Alpha-mannosidase (Q2R3E0)	9.53E-43
1567	CG – GT	TC57827	211	**-**	Galactose dehydrogenase, (Q84LI1)	4.13E-33
255	TA – AG	TC52686	210	**-**	Carbonic anhydrase (Q5NE20)	2.18E-39
1472	CT – GC	TC60916	581	**-**	Glyceraldehyde-3-phosphate dehydr. B subunit	1.61E-126
1205	CT – AT	TC62475	182	**-**	Lipase class 3-like (Q6K2K7)	6.71E-33
1689	TT – GG	TC53435	141	**-**	B-keto acyl reductase (O24479)	4.93E-24
670	CA – GA	TC62496	145	**-**	3-hydroxy-3-methylglutaryl coenzyme A (Q8W2E3)	6.52E-12
151	TC – TC	Q8H539	358	**-**	Steroid 5alpha-reductase-like protein	1.00E-32*
1239	CT – AG	TC69679	98	**-**	60S ribosomal protein L19 (Q6RYC4)	1.65E-13
138	TC – TT	TC59193	99	**-**	Protein translation factor SUI1 homolog 2 (Q94JV4)	1.49E-12
308	TA – CA	TC51783	621	**-**	Elongation factor 1-beta 1 (Q84WM9)	1.99E-64
823	TC – GG	TC54220	542	**-**	Phytocalpain (Q6SSJ2)	3.95E-39
1174	CC – CC	TC63107	115	**-**	Chaperone protein dnaJ-like (Q6H3Y3)	9.58E-18
1530	CC – GC	CB348741	62	**-**	F-box protein family AtFBL5	1.37E-12
1476	CT – GC	CA816379	345	**-**	Protein At3g07360 (U-box domain-containing prot.)	2.37E-69
1389	CG – TT	TC65574	203	**-**	Glycin-rich protein (Q43688)	3.13E-35
1546	CG – GA	TC68519	541	**-**	Expansin, complete (Q84US7)	8.40E-112
722	TC – CT	TC69230	350	**-**	Cyclin D1, partial (Q8GVE0)	1.73E-69
664	CA – GA	TC53870	228	**-**	Integral membrane protein-like (Q5VRH)	7.89E-07
**Photosyntesis and Energy**						
1330	CT – CC	TC61438	646	**+**	Cytochrome P450 monooxygenase (Q2MJ14)	1.38E-67
123	TC – TA	TC68921	64	**+**	C-type cytochrome biogenesis protein (Q7XY14)	1.08E-06
227	TA – AT	TC62259	353	**-**	ATP synthase B' chain, chloroplast precursor	1.03E-66
749	TC – CG	TC63430	230	**-**	NADPH-cytochrome P450 oxydoreductase	1.20E-29
520	TG – TG	TC65998	102	**-**	Plastocyanin, chloroplast precursor (P17340)	6.20E-06
1247	CT – TA	TC66316	365	**-**	Chloroplast photosystem II 10 kDa protein (Q2PXN6)	5.90E-58
1156	CC – CT	TC68056	154	**-**	NADH-plastoq. oxidoreductase subunit 7 (Q2L953)	2.39E-26
1049	CC – AC	TC53584	561	**-**	Magnesium chelatase subunit, partial	1.28E-121
31	TT – AT	TC55659	163	**-**	Chlorophyll A/B binding protein precursor (Q32291)	1.79E-32
427	TG – TA	TC57132	209	**-**	Chlorophyll A/B binding protein, precursor (P13869)	3.62E-42
4	TT – AA	TC55138	381	**-**	Photosystem I assembly protein ycf3, partial	2.10E-78
1115	CC – TC	TC61693	411	**-**	Photosystem I reaction center subunit II (P12372)	2.51E-84
1322	CT – CT	TC66994	275	**-**	Photosystem II reaction center (Q8W536)	2.26E-52
161	TC – TC	TC58978	146	**-**	Photosystem II M protein (Q6QXV8)	3.80E-25
1393	CG – TT	TC58567	103	**-**	Apocytochrome f precursor (Q68RZ3)	4.92E-10
**Transport**						
1614	TT – CA	Q3L7K6	185	**+**	Hexose transporter	6.26E-08 *
680	CA – GT	TC52346	231	**-**	Transporter-like protein (Q9LSH7)	3.88E-47
249	TA – AC	TC66367	107	**-**	14-3-3 protein (Q93XW1)	1.13E-16
1534	CC – GG	TC57372	435	**-**	Nitrate transporter NRT1-2 (Q9FRU5)	8.33E-94
1438	CG – CC	TC65826	270	**-**	ABC transporter-like protein (Q9FT51)	7.38E-50
133	TC – TT	TC62785	219	**-**	Calcium-transporting ATPase 8, plasma membr.	4.00E-23
662	CA – GA	TC69251	302	**-**	ATPase alpha subunit, complete (Q3C1H4)	7.47E-59
1679	TT – GC	BQ798655	127	**-**	Syntaxin {Glycine max}	5.39E-23
792	TC – GT	TC51721	180	**-**	ADP-ribosylation factor 1-like protein (Q70XK1)	2.22E-36
62	TT – AG	TC69827	195	**-**	Aquaporin PIP2 (Q2HZF5)	1.45E-20
**Signal transduction**						
434	TG – TA	TC59460	147	**+**	Protein phosphatase 2C (Q8RVG0)	6.61E-23
240	TA – AC	Q52QR5	343	**+**	NAC domain protein NAC1	1.70E-71*
1385	CG – TA	TC55407	95	**+**	NAC family protein (Q2Z1Y1)	2.32E-13
687	CA – GT	TC70344	687	**+**	WRKY DNA-binding protein 32 (P59583)	1.24E-05
235	TA – AT	TC52484	102	**-**	SOS2-like protein kinase (Q8LK24)	1.58E-12
1298	CT – TG	TC60214	164	**-**	Calcium-transporting ATPase/calmodulin binding	2.74E-28
288	TA – TC	TC52400	212	**-**	Calmodulin cam-207 (Q6DN29)	1.23E-39
1538	CC – GG	TC66781	267	**-**	CNGC2 (Q5D6H2)	1.22E-47
633	TG – GC	TC69501	633	**-**	Calcium sensor calcineurin B-like protein (Q4W3B4)	1.02E-34
11	TT – AA	TC61968	289	**-**	Protein At1g01140 (CBL-interacting protein kinase)	7.34E-54
778	TC – GA	TC53469	55	**-**	MAPKK (Q66MH6)	8.34E-07
1573	CG – GC	TC59576	700	**-**	Receptor-like kinase with LRR repeats (Q70AH8)	1.44E-32
1231	CT – AG	TC52043	339	**-**	MADS-box transcription factor FBP29 (Q9ATE2)	2.44E-72
**Secondary metabolism**						
1539	CC – GG	TC52853	419	**+**	Cinnamyl alcohol dehydrogenase (Q2Z1Z0)	2.21E-08
909	CA – AG	TC53668	105	**+**	Stilbene synthase [Vitis vinifera]	5.34E-17
1249	CT – TA	TC54354	273	**-**	Secretory laccase (Q6TDS6)	1.11E-23
421	TG – AG	TC51729	113	**-**	Caffeoyl-CoA O-methyltransferase (Q43237)	5.87E-17
362	TG – AA	TC53331	208	**-**	Flavanone 3-hydroxylase-like protein, (Q9FLV0)	2.88E-34
432	TG – TA	TC65435	179	**-**	Limonoid UDP-glucosyltransferase (Q9MB73)	1.29E-32
**Defence response**						
1176	CC – CG	TC63410	557	**+**	Beta-1,3-glucanase (Q9M563)	5.95E-120
68	TT – AG	TC56756	108	**+**	Pathogenesis-related protein 10.3 (Q20BD2)	6.31E-14
724	TC – CT	TC56512	253	**-**	Pollen allergen-like protein, partial {A. thaliana}	1.09E-49
1296	CT – TG	TC62916	157	**-**	EXECUTER1 protein (Q93YW0)	1.72E-27
692	CA – GC	TC57989	251	**-**	Avr9/Cf-9 rapidly elicited protein 276 (Q84QD7)	3.72E-23
229	TA – AT	TC70153	282	**-**	Flax-inducible sequence 1 (Q40255)	6.91E-56
1173	CC – CC	TC51855	262	**-**	Pto-like serine/threonine kinase (Q6W0C7)	1.69E-16
1545	CG – GA	TC58939	671	**-**	Ethylene-resp. element-binding factor (Q2QDF5)	3.13E-125
584	TA – GC	TC62111	83	**-**	Lipid transfer protein (Q93YX9)	4.49E-10
889	CA – AC	TC63540	305	**-**	DIR1 (At5g48485) {Arabidopsis thaliana}	2.98E-58
214	TT – TA	TC61755	233	**-**	Enzymatic resistance protein, complete (Q3S4G9)	1.61E-44
**Response to stimulus**						
246	TA – AC	TC63756	153	**+**	Metallothionein-like protein (Q3HR41)	3.69E-27
945	CA – TT	TC53817	79	**+**	Thioredoxin H, (Q4U0W0)	1.68E-08
1275	CT – TC	TC53088	383	**+**	Glutathione S-transferase GST 24 (Q9FQD4)	7.96E-81
748	TC – CG	TC54876	240	**+**	Ferritin-3, chloroplast precursor (Q948P6)	2.59E-47
1631	TT – CT	Q6V7W6	84	**+**	Class III peroxidase GvPx2b	2.76E-04
1245	CT – TA	TC62299	380	**-**	Auxin-induced SAUR-like protein (Q8S351)	1.89E-79
1562	CG – GT	TC53184	397	**-**	Chloroplast small heat shock protein (Q6WHC0)	1.14E-80
781	TC – GA	TC53791	232	**-**	Catalase (Q7XTK9)	4.25E-45
532	TG – CT	TC56223	206	**-**	Peroxiredoxin Q (Q6QPJ6)	4.38E-37

I/R: induced or repressed in cDNA-AFLP experiments.

### Identification of *P. viticola *genes expressed in grapevine during infection

Because there is little data on *P. viticola *virulence factors released by the pathogen during infection, the identification of upregulated transcripts and their cross-reference to known oomycete genes was an important goal of this study. Databases containing genomic sequence information from *Phytophthora sojae*, *P. ramorum*, *P. infestans *and *Hyaloperonospora parasitica *were used for comparison. We identified 96 TDFs expressed *in planta *during infection that could be attributed to *P. viticola *based on their similarity to other oomycete sequences, 22 corresponding to non-annotated genomic contigs and 74 with functional annotations, including genes involved in protein and lipid metabolism, signal transduction, transport, response to oxidative stress and toxicity (Table 2 and Additional File 2).

**Table 2:** List of selected putative *P. viticola *TDFs expressed *in planta*

**TDF**	**Primer Comb**.	**Lenght (bp)**	**Homology**	**Annotation**	**Blast score**
170	TC – TG	213	Ps_004_22448_Jun03	PROBABLE 50S RIBOSOMAL PROTEIN L1 [Sinorhizobium meliloti]	6.00E-05
1272	CT – TT	72	Pi_006_52843_Feb05	60S ribosomal protein L11 [Hyacinthus orientalis]	2.00E-05
1214	CT – AC	400	Ps_060_22857_Jun03	60S RIBOSOMAL PROTEIN L7A protein [Arabidopsis thaliana]	7.00E-77
1390	CG – TT	114	Pi_001_74169_Feb05	RL35_EUPES 60S ribosomal protein L35	8.00E-09
1421	CG – CA	64	Pi_015_57096_Feb05	RL9_SPOFR 60S ribosomal protein L9	6.00E-05
1565	CG – GT	216	Pi_004_46349_Feb05	L-aspartate oxidase [Chromobacterium violaceum ATCC 12472]	1.00E-05
1132	CC – TG	89	Pi_001_82736_Feb05	Nascent polypeptide associated complex alpha chain prot. [A. thaliana]	6.00E-12
862	CA – AA	171	Pi_030_51789_Feb05	Polyubiquitin [Plasmodium falciparum 3D7]	5.00E-20
1228	CT – AC	89	Ps_016_22726_Jun03	Ubiquitin-conjugating enzyme e2-16 kd [S. pombe]	6.00E-09
1135	CC – TG	81	Pi_003_44814_Feb05	Actin depolymerizing factor – like protein [A. thaliana]	5.00E-09
1484	CT – GC	127	Pi_002_46251_Feb05	Fructose-1 6-biphosphatase [Phytophthora infestans]	5.00E-04
160	TC – TC	174	Ps_016_22911_Jun03	Myo-inositol-1-phosphate synthase [Nicotiana paniculata]	2.00E-22
83	TC – AC	240	Ps_005_22630_Jun03	Putative succinate dehydrogenase flavoprotein subunit [A. thaliana]	4.00E-12
394	TG – AC	277	Ps_001_26384_Jun03	Putative steroid binding protein [A. thaliana]	1.00E-12
241	TA – AC	288	Pi_004_52230_Feb05	Fatty acid synthase, subunit alpha – fission yeast (S. pombe)	1.00E-31
1604	TT – TG	81	Ps_029_22780_Jun03	14-3-3 protein epsilon [Xenopus laevis]	3.00E-07
875	CA – AT	203	Pi_003_45566_Feb05	ATPase, H+ transporting [Gallus gallus]	6.00E-14
1366	CG – AG	129	Ps_001_28794_Jun03	Putative beta-subunit of K+ channels [Solanum tuberosum]	5.00E-07
689	CA – GC	422	Ps_018_22812_Jun03	Mn superoxide dismutase [Chlamydomonas reinhardtii]	1.00E-105
542	TG – CG	170	Pi_018_51411_Feb05	Mitochondrial Mn3+ Superoxide Dismutase	4.00E-30
794	TC – GT	256	Ps_040_22917_Jun03	Homology to G protein beta subunit [Chlamydomonas reinhardtii]	1.00E-21
1410	CG – TG	289	Pi_005_48934_Feb05	C3HC4-type RING zinc finger protein-like [Oryza sativa ]	5.00E-12
270	TA – TA	182	Pi_009_51114_Feb05	GTP-binding protein ora3 – [Gallus gallus]	5.00E-05
239	TA – AC	92	Pi_001_76149_Feb05	NADH2 dehydrogenase (ubiquinone) [Canis familiaris]	1.00E-13
292	TA – TC	87	Pi_004_51216_Feb05	Probable atrazine chlorohydrolase [Chromobacterium violaceum ]	3.00E-07
1274	CT – TC	397	Pi_001_66405_Feb05	Putative citrate synthase [Saccharomyces kluyveri]	5.00E-81
552	TA – GA	222	Pi_002_43637_Feb05	Putative nuclear LIM interactor-interacting prot. [Phytophthora sojae]	3.00E-10
1010	CA – CC	521	Pi_003_48725_Feb05	S28245 NADH2 dehydrogenase (ubiquinone)	2.00E-34
208	TT – TA	64	Pi_004_48444_Feb05	T52339 ADP-ribosylation factor – pepper	6.00E-11
826	TC – GG	291	Pi_028_48244_Feb05	Vacuolar H+-pyrophosphatase [Prunus persica]	2.00E-58

We predicted that 87 of the *P. viticola*-derived fragments were of pathogen origin because similar-sized bands were present in the sporangia cDNA-AFLP lanes. The remaining nine fragments were not present in sporangia, and were thus uniquely induced by infection. These are the most likely to represent putative virulence factors. However, alignment to a database of putative *avr *homologs, kindly provided by Dr. J. Win, Sainsbury Laboratory (UK), failed to identify any *P. viticola*-derived fragments with similarity to predicted *Phytophthora *effectors.

### Verification of representative genes by real-time RT-PCR

**Figure 3:**
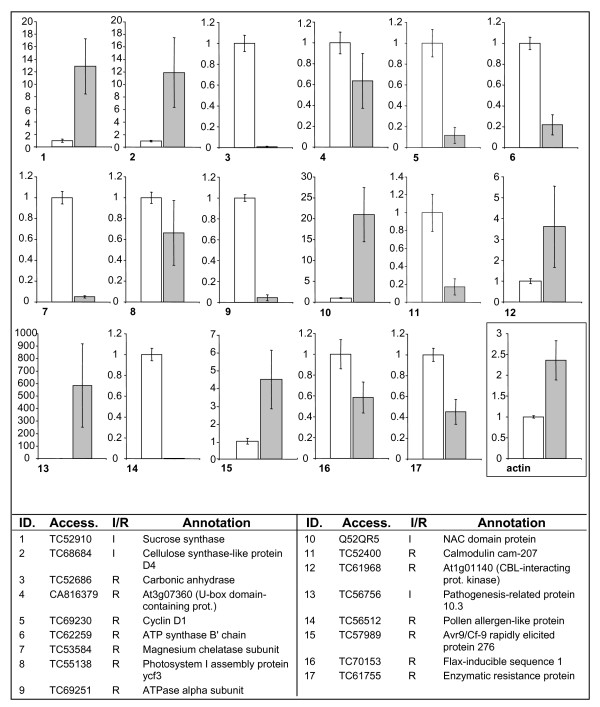
**Real-time RT-PCR analysis**. Real-time RT-PCR analysis of transcript levels for 17 selected genes in healthy (white) and infected (gray) grapevine leaves. Gene annotations correspond to numbers on graphs. I/R: induced or repressed according to cDNA-AFLP experiments. The expression level of actin (TC60835) is also reported (insert). All data were normalized to the 18S rRNA expression level. Data represent fold change of gene expression in infected vs. control samples. Bars represent a 95% confidence interval, calculated on 3 technical replicates.

The expression level of 17 modulated grapevine transcripts was analyzed further by real-time RT-PCR, to validate cDNA-AFLP expression profiles. These genes were chosen as they represented almost all the different functional categories identified, with a preference for defense-related genes and genes possibly involved in pathogenesis. Results are shown in Figure 3. The choice of a stable reference gene for data normalization is still controversial in grapevine. Several genes that are commonly considered to be constitutively expressed, such as tubulin, ubiquitin and glyceraldehyde 3-phosphate dehydrogenase, were shown to be modulated in our experiments and in previous work [35]. Data were therefore normalized against 18S RNA expression levels, which are among the more stably-expressed genes in other oomycete infections [36]. Actin (TC60835), which was considered as a possible reference gene initially, also appeared to be modulated in the infected sample (Figure 3). The expression of the selected genes was in good agreement with profiles detected by cDNA-AFLP, except for two genes: TC57989 (avr9/Cf9 rapidly induced protein 276) and TC61968 (Protein At1g01140). These experiments allowed the detection of strong changes in gene expression (about 10-fold or more) between infected and healthy tissues for nine of the selected genes. Among defense-related transcripts, it is worth noting the ~600-fold increase in the level of mRNA for the homologue of pathogenesis-related protein 10.3 of *Vitis quinquangularis *(TC56756) (Figure 3, gene 13).

## Discussion

Transcriptomics is a powerful approach for the global analysis of plant-pathogen interactions, and our study used this strategy to provide the first large-scale investigation of the compatible interaction between *P. viticola *and grapevine. We observed widespread modulation of transcriptional activity, with 17% of all transcripts showing some form of differential expression, consistent with the extensive physiological changes that affect most cells in infected tissues.

The cDNA-AFLP method for global transcriptional analysis is an open architecture technology that is appropriate for gene expression studies in non-model species. This is because prior sequence data is not required for the visual identification of differentially-expressed transcripts, in contrast to other approaches. In addition, cDNA-AFLP is particularly useful for the study of plant-pathogen interactions because the method facilitates gene discovery in both organisms simultaneously [25,37,38]. These advantages are emphasized by our discovery that 25% of our TDFs representing modulated grapevine transcripts were not previously reported in any EST database. Because of the very small number of *P. viticola *sequences deposited in databases thus far, all putative *P. viticola *transcripts expressed *in planta *identified in this work could be regarded as newly identified genes.

### Infection with *P. viticola *causes widespread gene repression in grapevine

The most striking discovery in our investigation was that nearly 70% of the differentially-expressed grapevine genes we identified were downregulated during infection, possibly reflecting the exploitation of cellular resources and/or the suppression of defense responses [7]. At the oil spot stage, infection has already been established but the host cells must be kept alive to supply the pathogen with nutrients and to facilitate sporulation. This closely mirrors the early stages of natural infections, thus the activation of a non-specific senescence program seems unlikely. Additionally, most of the visualized transcripts were unaffected by infection, and 30% of the differentially-expressed genes were clearly upregulated confirming the absence of a general, global, repressive environment. Among the upregulated genes, we identified many usually considered to have "housekeeping" functions, such as a tubulin alpha chain (TC65238), a dynein light chain (TC57042) and, according to the real-time RT-PCR experiments, actin. The induction of a plant actin gene was first reported in *Malva pulsilla *during the biotrophic phase of interaction with *Colletotrichum gloesporioides *[39] and several subsequent reports supported actin's role in cytoskeleton rearrangement during incompatible interactions as well as in the maintenance of compatibility [40]. According to cytological data, it is also unlikely that the lower steady state mRNA levels could be due to the proportional increase in pathogen-derived transcripts in the mixture, as could be the case with hemibiotrophic or necrotrophic pathogens in late stages of infection [27]. In extensively colonized tissues, only apical parts of the mycelium seem to be metabolically active while older portions are totally devoid of cytoplasm [41,42]. Thus, the presence of pathogen RNA should not significantly reduce the amount of plant RNA compared to non-infected leaves. For these reasons, our data probably reflect the actual changes in mRNA levels that characterize this strictly biotrophic plant-microbe interaction. Data provided by real-time RT-PCR confirmed the original expression profiles for 15 out of 17 selected genes, further strengthening the reliability of our results.

#### Photosynthesis and primary carbon metabolism

The most striking transcriptional downregulation in our investigation was observed in genes related to photosynthesis, e.g. chlorophyll a-b binding proteins (TC54828, TC55242, TC56895) and photosystem components (TC53444, TC61693, TC66994), consistent with the measurable reduction in chlorophyll content during pathogenesis [43]. Transcriptional downregulation of photosynthesis-related genes has been reported previously during compatible interactions between potato and *P. infestans *[28] and between soybean and *P. sojae *[27]. Similar results for grapevine have been reported in microarray-based analyses of compatible interactions with viruses and powdery mildew [44,45]. It is well established that plants infected with biotrophic fungal pathogens, such as powdery mildews and rusts, reduce their photosynthetic rates, possibly as a result of increased invertase activity which causes carbohydrate accumulation [46]. Invertase is needed to cleave sucrose into hexose sugars, which in turn can be taken up by pathogens. In this context, the increased level of two genes with similarity to hexose transporters (Q3L7K6 and TC66367) is also worth noting. Carbohydrate accumulation may inhibit the Calvin cycle, which also limits photosynthesis [46,47]. Several genes encoding enzymes in the Calvin cycle are downregulated during infection, among them ribulose bisphosphate carboxylase/oxygenase activase (TC66665) a plastidic aldolase (TC52159), a sedoheptulose bisphosphatase (TC54570), a phosphoribulokinase (TC56646), and a plastidial glyceraldehyde-3-phosphate dehydrogenase B subunit (TC60916).

*P. viticola *infection also elevated mRNA levels for a sucrose synthase (TC52910), an enzyme that usually carries out sucrose degradation in plants. This reaction releases fructose and UDP-glucose residues, which are substrates for callose and cellulose synthesis. Therefore it is interesting to note that two UDP-glucosyltransferases (TC57852 and TC54299) are also among the upregulated transcripts we identified, along with a cellulose synthase-like sequence (TC68684). Moreover, since cellulose and callose are the main components of *Plasmopara *cell walls and septa, we speculate that the induction of these genes might reflect the supply of precursors for pathogen metabolism. Concomitantly, several genes encoding cell wall degrading enzymes are downregulated, including two pectinacetylesterases (TC54500 and TC52435) and a polygalacturonase-like protein (TC59719).

The carbonic anhydrase (CA) gene TC52686 is worth particular attention, as its downregulation during infection was established by both cDNA-AFLP and real-time RT-PCR. In C4 plants, CA catalyzes the reversible hydration of carbon dioxide to bicarbonate and provides carbon dioxide for fixation by RuBisCO. However, the role of CA in C3 plants, such as grapevine, is less clear [48,49]. The enzyme has antioxidant activity and is known to bind salicylic acid [50]. It is downregulated in tomato plants following application of the fungal toxin fusicoccin [51], in *Arabidopsis *following treatment with methyl jasmonate [52] and in potato infected with *P. infestans *[28]. Silencing of CA expression in *Nicotiana benthamiana *resulted in suppression of the *Pto*:*avrPto-*mediated hypersensitive response [50] and in increased susceptibility to *P. infestans *[28]. Taken together, these data suggest CA could be involved in the pathogen response and/or that downregulation of CA could be required for the maintenance of a compatible interaction.

#### Lipid metabolism

Lipid-derived molecules act as signals in plant-pathogen interactions, with jasmonic acid (JA) and related oxylipins produced from membrane-derived fatty acids through beta-oxidation, having particularly important roles [53]. Lipid accumulation is usually associated with necrogenic infections and insect infestations, but JA could also be involved in resistance against biotrophic pathogens, as suggested in grapevine for BABA-induced resistance to *P. viticola *[54]. During infection, low level defense responses can be activated in susceptible plants, as already reported in grapevine [21,45,55]. Therefore, it is not surprising that well-established *P. viticola *infections involve the upregulation of genes encoding different enzymes in the beta-oxidation pathway, such as two 3-ketoacyl-CoA thiolases (TC53311 and TC55776), an acyl-coenzyme A oxidase (TC58112) and a fatty acid multifunctional protein (TC52362), as well as a gene encoding a 12-oxophytodienoate reductase (TC67104) that could be involved in the metabolism of oxylipin signaling molecules. Fatty acid metabolism can also produce aldehydes and alcohols with antimicrobial properties, a process involving lipoxygenases and hydroperoxide lyases, examples of which were also induced by infection (CF074703 and TC55722). Other lipases were repressed during infection (DT013748, Q6K832 and TC62475). Certain genes involved in sterol biosynthesis were induced (3-beta-hydroxy-delta5-steroid dehydrogenase, TC67959) while others were repressed (steroid-5-alfa-reductase like protein, Q8H539). Because *P. viticola *appears fully dependent on its host for sterol biosynthesis [56,57], the modulation of transcripts involved in the sterol biosynthesis pathway needs to be investigated in more detail.

#### Protein metabolism

Genes related to protein metabolism were also prevalently repressed in our experiment. Among them were genes encoding ribosomal proteins, protein modification and degradation enzymes (e.g. ubiquitin-conjugating enzymes), as well as several kinases, phosphatases and peptidases, which could also be involved in intracellular and intercellular signaling. This suggested a general repression of protein synthesis and turnover. However, some genes involved in amino acid biosynthesis were induced, such as a cysteine synthase (TC51806) and a gamma-glutamylcysteine synthetase (TC56558), in agreement with previous findings [7].

#### Transport

About 7% of the modulated transcripts corresponded to genes involved in transport. This probably reflects the peculiar nutritional strategy of oomycetes, which rely on molecular trafficking through a modified plasma membrane with inactivated ATPases [58]. We observed the downregulation of genes encoding five different membrane ATPases (TC62785, TC53387, TC69251, TC58445, TC60214), as well as 14-3-3 proteins (TC52346, TC54584), proteins related to vesicular traffic (BQ798655) and ABC transporters (TC57412, TC65826). Genes encoding amino acid and hexose transporters were upregulated perhaps to facilitate the transfer of nutrients to the pathogen (Q1SRS8, Q3L7K6, TC66367, TC62234).

#### Signal transduction

About 14% of the modulated genes had signal transduction and/or gene regulation functions, including two WRKY DNA-binding proteins (TC70344, Q1T4J9) [59], two NAC transcription factors (TC55407, Q52QR5) [60] and a phosphatase 2C (TC59460) which were induced by infection. However, the majority of genes in this category were downregulated. Several genes encoding components of the calcium signaling network were among them, including calmodulin (TC52400), calmodulin-binding proteins (TC59357, TC68333), a calcium sensor calcineurin B-like protein ('TC69501) and a calcium-dependent protein kinase (CF211026). Calcium signaling is known to be essential in some plant defense mechanisms [61,62]. Many other signaling components and transcription factors were repressed, suggesting that the suppression of endogenous signaling pathways is required to establish compatible interactions.

#### Secondary metabolism, defense and responses to external stimuli

Many plant defense responses involve the production of secondary metabolites [63]. In the secondary metabolism category, we found that about the same number of genes were upregulated and downregulated, in contrast to all other functional categories. For example, phenylpropanoid pathway enzymes are necessary for the biosynthesis of antimicrobial phenolic derivatives, lignanes and phytoalexins. Several genes encoding enzymes in this pathway were upregulated in infected leaves, including a caffeoyl-CoA O-methyltransferase (TC51729), a stilbene synthase (TC53668), a secretory laccase (TC54354), as well as two glucanases (TC63410, TC60651) and a pathogenesis-related protein 10.3 (TC56756). This indicates the presence of a general although weak defense response in susceptible plants. In contrast we identified homologs of a Pto-like serine/threonine kinase (TC51855) [64], the enzymatic eR protein (TC61755) [65] and the resistance protein KR4 (BQ800016) [66] all of which were downregulated. This was also the case for lipid transfer proteins, such as two homologues of the DIR1 gene (TC63540 and TC61952) [67], a homolog of the Avr9/Cf-9 rapidly elicited protein 276 [68], and a homolog of the *Arabidopsis EXECUTER-1 *gene (TC62916) [69]. All these genes have been assigned a function related to resistance in other pathosystems, and will be subject to further investigations. Additional genes, that respond to a variety of external stimuli and are often involved in the control of redox balance in the cell, were prevalently downregulated during infection, such as a catalase (TC53791) and a peroxiredoxin Q (TC56223).

### *P. viticola *genes expressed *in planta*

The exclusively biotrophic lifestyle of *P. viticola *and other oomycetes complicates the identification of pathogenicity factors. Even so, we identified 96 TDFs corresponding to *P. viticola *transcripts expressed *in planta *and nine of these sequences appeared to be expressed at detectable levels only in infected tissues, not in sporangia, even though RNA from the pathogen is much more abundant in the sporangia. These nine transcripts therefore represent important candidate genes specific for the infection process.

Several *P. viticola *transcripts were homologous of genes involved in protein metabolism. Shan and colleagues [70] showed that several 60S ribosomal protein subunits are expressed at the onset of infection with *Phythopthora nicotianae*, indicating a requirement for protein synthesis in the pathogen. Several *Plasmopara *transcripts are homologous to enzymes involved in carbohydrate and fatty acid metabolism, in energy production, and in cellular transport. Genes encoding anti-oxidant enzymes, such as a homolog of *P. nicotianae *manganese superoxide dismutase [71], proteins involved in signal transduction such as a homolog of *Chlamydomonas *beta-subunit-like polypeptide CBLP [72] and a steroid binding protein [73] are also expressed in the interaction. All these aspects deserve further investigation in the light of their importance in fungal pathogenesis.

A large repertoire of virulence effectors is thought to be secreted by oomycete pathogens in order to manipulate their host cells [9]. Several approaches have been used to identify such factors in different plant-oomycete interactions [7,9,26,27,29,30]. Following the discovery of a conserved motif (RXRL-EER) necessary for translocation to the plant in all known oomycete avirulence proteins [12,14], bioinformatic tools have been applied to search for putative effector proteins in the different *Phytophthora *sequenced genomes [13]. This led to the identification of about 700 putative avirulence genes, but none of the identified *P. viticola *TDFs showed any similarity to predicted *Phytophthora *effectors.

## Conclusion

This report describes the first large-scale investigation into the molecular basis of compatibility between *Vitis vinifera *and the strictly biotrophic pathogen *Plasmopara viticola*. The cDNA-AFLP technique allowed the discovery of novel genes both in grapevine and in *P. viticola*, as a significant proportion of TDFs are not currently represented in *Vitis *or in oomycete EST databases.

Our data show that infection results primarily in the downregulation of grapevine transcripts in all major functional categories, especially photosynthesis. However, certain genes required for plant-pathogen interactions are positively modulated during infection at the oil spot stage. Actin was also upregulated in infected leaves, reflecting the occurrence of important cytoskeleton modifications during downy mildew infection, and further indicating that assumption of constitutive expression for "housekeeping" genes must always be considered with caution in specific physiopathological conditions. This work also provides the largest available repertoire of *P. viticola *genes expressed *in planta*. A large amount of information concerning mRNA levels in infected grapevine is now available, which will hopefully serve as a basis to address new questions and design new experiments to elucidate further the biology of plant-oomycete interactions and the associated re-programming of host metabolism.

## Methods

### Plant material, inoculum and pathogen infection

Grapevine plants (cv Riesling) were grown in greenhouse at 19°C with 70–80% relative humidity. *P. viticola *inoculum was collected from sporulated field leaves and used for the artificial inoculations of surface-sterilized leaves. The inoculum was stored as sporangia at -20°C. Infections were initiated by spraying the third and fourth grapevine leaves with a suspension of 10,000 sporangia per ml in cold pure water. The leaves were covered for one night with plastic bags to increase humidity and the plants were kept in the same greenhouse at 19°C with a 16-h photoperiod. Within 2–3 weeks, infected leaves developed the typical oil spot symptoms. The oil spot lesions were sampled with a cork-borer and used for RNA extraction. As a control, RNA was extracted from water-treated leaves incubated under the same conditions. RNA extraction from leaves has been described [74]. Total RNA from sporangia was extracted from frozen samples (50–100 mg) with the RNeasy Mini Kit (Qiagen) following the manufacturer's protocol for plant tissues.

### cDNA-AFLP analysis

The cDNA-AFLP protocol applied [31] is a modification of the original technique [32] which permits the visualization of one single cDNA fragment for each messenger originally present in the sample, thus reducing the redundancy of sequences obtained. Briefly, double-stranded cDNA was synthesized from 2 μg total RNA using the Superscript II reverse transcription kit (Invitrogen) and a biotinylated oligo-dT primer (Promega). The cDNA was digested with *Bst*YI (restriction site RGATCY), and the 3' ends of the fragments were captured on streptavidin magnetic beads (Dynal). Digestion with *Mse*I released yielded fragments that were ligated to adapters for amplification (*BstYI*-Forw: 5'-CTC GTA GAC TGC GTA GT-3'; *BstYI*_Rev: 5'-GAT CAC TAC GCA GTC TAC-3'; *MseI-*Forw: 5'-GAC GAT GAG TCC TGA G-3'; *MseI-*Rev: 5'-TAC ATC AGG ACT CAT-3'). Pre-amplification was performed with an *Mse*I primer (Mse0: 5'-GAT GAG TCC TGA GTA A-3'), combined with a *Bst*YI primer carrying either a T or a C at the 3' end (BstT0: 5'-GAC TGC GTA GTG ATC T-3'; BstC0: 5'-GAC TGC GTA GTG ATC C-3'). Pre-amplification PCR conditions were as follows: 5 min denaturation at 94°C and then 30 s denaturation at 94°C, 60 s annealing at 56°C, 60 s extension at 72°C (25 cycles), followed by 5 min at 72°C.

After preamplification, the mixture was diluted 600 fold and 5 μl was used for selective amplification with 128 primer combinations, carried out with one selective nucleotide added on the ^33^P-labeled *Bst*YI primer and two selective nucleotides on the *Mse*I primer. Touch-down PCR conditions for selective amplifications were as follows: 5 min denaturation at 94°C, followed by 30 s denaturation at 94°C, 30 s annealing at 65°C, 60 s extension at 72°C (13 cycles, scaledown of 0.7°C per cycle); 30 s denaturation at 94°C, 30 s annealing at 56°C, 60 s extension at 72°C (23 cycles) and 5 min at 72°C. Selective amplification products were separated on a 6% polyacrylamide gel in a Sequi-Gen GT Sequencing Cell (38 × 50 cm) (Bio-Rad) running for 2.5 h at 105 W and 50°C. Gels were dried onto 3 MM Whatman paper on a Gel Dryer Model 583 (Bio-Rad) and marked with Glogos II Autorad Markers (Stratagene) before exposing to Kodak Biomax MR films, for 24 h. The bands of interest were cut from the gels with a surgical blade and eluted in 100 μl of sterile distilled water. An aliquot of 5 μl was used as a template for reamplification using non-labeled primers identical to those employed for selective AFLP amplification. PCR products were purified with MultiScreen PCR μ96 plates (Millipore) and sequenced directly (BMR Genomics).

### Sequence analysis

Homology searching by BLAST [75] was carried out against the following databases: NCBI [76] DFCI Grape Gene Index [77], Genoscope Grape Genome database [78], UNIPROT [79], PFGD Phytophthora Functional Genomics Database [80] and VBI Microbial Database [81]. Sequences were manually assigned to functional categories based on the analysis of scientific literature and also with the aid of the information reported for each sequence by The Gene Ontology Consortium [34], when available, or reported by the Swiss-Prot [82], KEGG [83] and TAIR [84] databases.

### Real-time RT-PCR analysis

Real-time RT-PCR was carried out on RNA derived from two independent biological experiments. Each sample was a pool of identical quantities of RNA from the two experiments. All samples were examined in three technical replicates. First-strand cDNA was synthesized from DNase-treated total RNA using "High Capacity cDNA Reverse Transcription Kit" (Applied Biosystems). Specific primer pairs (20 b) were designed on 17 TDFs (Additional file 3) and tested by RT-PCR. Primers specific for *Vitis vinifera *18S rRNA were used for the normalization of reactions. Experiments were carried out using Power SYBR Green PCR Master Mix (Applied Biosystems) in a Mx3000P QPCR Systems (Stratagene). The following thermal cycling profile was used: 95°C 10 min; 45 cycles of 95°C for 30 s, 58°C for 30 s, 72°C for 30 s; 95°C for 1 min, 55°C for 30 s, 95°C for 30 s. Each real-time assay was tested in a dissociation protocol to ensure that each amplicon was a single product. Data were analyzed using MxPro QPCR software (Stratagene). The LinRegPCR software [85] was used to confirm that PCR efficiency was about 2 for each primer couple, and 18S rRNA expression was used as an internal control to normalize all data. Fold change in RNA expression was estimated using threshold cycles, by the ΔΔC_T _method [86].

## Abbreviations

cDNA: Complementary DNA; DFCI: Dana-Farber Cancer Institute; EST: Expressed Sequence Tag; NCBI: National Center for Biotechnology Information; TAIR: The *Arabidopsis *Information Resource; TDF: Transcript-derived fragment; VBI: Virginia Bioinformatics Institute.

## Authors' contributions

MP carried out the cDNA-AFLP experiments (including the extraction and reamplification of cDNA fragments) participated in sequence analysis, performed the real-time RT-PCR experiments, and contributed to data interpretation and manuscript writing. FD and AZ participated in reamplification of cDNA fragments, in real-time RT-PCR experiments and in the analysis and interpretation of data. AF participated in sequence analysis, in automatic Gene Ontology assignment and to table editing. MPz participated in experiment supervision, interpretation of data and critical reading of the manuscript. AK performed *P. viticola *infections, RNA extractions from sporangia and infected plant material, participated in experiment design and coordination, in interpretation of data, and in writing the manuscript. AP conceived the study, participated in its design and coordination, participated in interpretation of the data, in manual ontology assignments and wrote the manuscript. All authors read and approved the final manuscript.

## Supplementary Material

Additional file 1 Complete list of grape transcripts modulated in *P. viticola *infected leaves. all genes are grouped in functional categories, according to the presentation in the paper. Click here for file

Additional file 2 Complete list of transcripts attributed to *P. viticola *and expressed in infected grape leaves. Click here for file

Additional file 3 List of the primers used for real-time RT-PCR experiments. Click here for file
